# Pleurocidin Peptide Enhances Grouper Anti-*Vibrio harveyi* Immunity Elicited by Poly(lactide-co-glycolide)-Encapsulated Recombinant Glyceraldehyde-3-phosphate Dehydrogenase 

**DOI:** 10.3390/vaccines2020380

**Published:** 2014-05-14

**Authors:** Shu-Chun Chuang, Wan-Ling Huang, Sau-Wei Kau, Yun-Pei Yang, Chung-Da Yang

**Affiliations:** 1 Department of Physiology, College of Medicine, Kaohsiung Medical University, No. 100, Shih-Chuan 1st Road, Kaohsiung 807, Taiwan; E-Mail: f86225016@ntu.edu.tw; 2 Graduate Institute of Animal Vaccine Technology, National Pingtung University of Science and Technology, No. 1, Shuefu Road, Neipu, Pingtung 912, Taiwan; E-Mails: e09011lisa@yahoo.com.tw (W.-L.H.); ansme5566@gmail.com (S.-W.K.); m2304223@yahoo.com.tw (Y.-P.Y.)

**Keywords:** *Vibrio harveyi* (*V. harveyi*), pleurocidin (PLE), glyceraldehyde-3-phosphate dehydrogenase (GAPDH), poly(lactide-co-glycolide) (PLG)

## Abstract

Outer membrane proteins, such as glyceraldehyde-3-phosphate dehydrogenase (GAPDH), are considered immunodominant antigens for eliciting protective immunity against *Vibrio harveyi*, the main etiological agent of vibriosis in fish. Cationic antimicrobial peptides (AMPs), such as pleurocidin (PLE), play important roles in activating and recruiting immune cells, thereby contributing to subsequent innate and adaptive immune responses. In the present study, we aimed to use PLE peptide as a potent adjuvant to improve the immunogenicity of *V. harveyi* recombinant GAPDH (rGAPDH). In order to prepare a controlled-release vaccine, PLE peptide and rGAPDH protein were simultaneously encapsulated into polymeric microparticles made from the biodegradable poly(lactide-co-glycolide) (PLG) polymer. The resulting PLG-encapsulated PLE plus rGAPDH (PLG-PLE/rGAPDH) microparticles, 3.21–6.27 μm in diameter, showed 72%–83% entrapment efficiency and durably released both PLE and rGAPDH for a long 30-day period. Following peritoneal immunization in grouper (*Epinephelus coioides*), PLG-PLE/rGAPDH microparticles resulted in significantly higher (*p* < 0.05, nested design) long-lasting GAPDH-specific immunity (serum titers and lymphocyte proliferation) than PLG-encapsulated rGAPDH (PLG-rGAPDH) microparticles. After an experimental challenge of *V. harveyi*, PLG-PLE/rGAPDH microparticles conferred a high survival rate (85%), which was significantly higher (*p* < 0.05, chi-square test) than that induced by PLG-rGAPDH microparticles (67%). In conclusion, PLE peptide exhibits an efficacious adjuvant effect to elicit not only improved immunity, but also enhanced protection against *V. harveyi* in grouper induced by rGAPDH protein encapsulated in PLG microparticles.

## 1. Introduction

Grouper, a farmed fish species with high economic value, has rapidly become an important high-quality agricultural product in Southeast Asia. However, the intensification of the grouper aquaculture has also been accompanied by the outbreaks of infectious diseases. *Vibrio harveyi* is a major Gram-negative bacterial pathogen that causes serious vibriosis, resulting in massive death of grouper and dramatic economic loss [1]. Although antibiotics have been used to control vibriosis in grouper, they give rise to serious drawbacks, such as the emergence of resistant bacterial strains and drug residues in fish [2]. Vaccination with effective vaccines is therefore an alternative, safer strategy for controlling vibriosis in fish. 

Sommerset *et al.* have indicated that effective vaccines comprising immunodominant bacterial subunits can elicit stronger protection in fish than inactivated whole-cell bacteria [3]. Current development efforts of subunit vaccines against *V. harveyi* have been focused mainly on the outer membrane proteins, such as glyceraldehyde-3-phosphate dehydrogenase (GAPDH), since antigenic epitopes on the bacterial surface are favorably accessible to the host immune system [4,5,6,7]. Generally, GAPDH is an important enzyme in classical cytosolic glycolysis. However, it also performs other functional activities in different cell compartments [8]. In particular, the surface GAPDH of *V. harvey*i can display its immunogenicity to induce protective immune responses in fish [6,7]. Therefore, GAPDH can be considered a proper antigen candidate for the development of an efficacious anti-*V. harveyi* vaccine. If alternative potent adjuvants that can effectively enhance the immunogenicity of a vaccine antigen [9,10,11], such as GAPDH, are used in the vaccine formulation, improved anti-*V. harveyi* protective immunity may be achieved in fish. 

Antimicrobial peptides (AMPs) are small cationic peptide molecules that have been identified and isolated from various species of organisms [12]. Generally, AMPs not only inhibit the growth of a broad spectrum of microbes through membrane disruption [13], but also participate in regulating innate and adaptive immune responses [10,14,15]. Pleurocidin (PLE), a cationic 25-residue AMP, is found in the skin-secreted mucous fluid of winter flounder (*Pleuronectes americanus*) [16,17]. *In vitro* study in trout macrophages has demonstrated that PLE peptide is able to trigger the expression of immune-relevant genes encoding IL-1β and cyclooxygenase-2, which are critical modulators in activating innate immunity [18]. PLE peptide also effectively stimulates mast cells to produce proinflammatory chemokines, monocyte chemotactic protein-1 (MCP-1) and macrophage inflammatory protein-1β (MIP-1β) [19]. Such PLE-induced chemokines are capable of activating and recruiting immune cells, including neutrophils, monocytes, macrophages and T-lymphocytes, to the sites of tissue injury, infection and inflammation, thereby contributing to subsequent innate and adaptive immune responses [19]. The immunomodulatory activities mentioned above therefore make PLE peptide an attractive agent to be explored as an adjuvant in animal vaccines [20]. 

In its natural form, PLE peptide is highly susceptible to proteolytic degradation and then exhibits a short half-life in animals [17,21]. In order to overcome this issue, some modifications, such as amidation at the carboxyl terminus of PLE peptide, have been done to reduce the susceptibility to enzyme degradation [22,23]. In addition, the microparticles made from biodegradable and biocompatible poly(lactide-co-glycolide) (PLG) polymers have become efficacious drug delivery systems in recent years to not only protect drugs, including proteins or peptides, from unfavorable proteolytic degradation, but also to allow the sustained release of drugs over a long period [24,25]. PLG microparticles also promote the uptake of encapsulated proteins via antigen-presenting cells (APCs) [26] and then favorably elicit specific cell-mediated immunity [11]. 

In the present study, we first cloned the *GAPDH* sequence of *V. harveyi* to produce a recombinant GAPDH (rGAPDH) protein with a molecular weight of 37 kDa. In order to enhance the immunogenicity of rGAPDH, PLE peptide with amidation at its carboxyl terminus was used as a potent adjuvant to mix with rGAPDH protein. Afterwards, the mixture was encapsulated with the PLG polymer to prepare controlled-release PLG-encapsulated PLE plus rGAPDH (PLG-PLE/rGAPDH) microparticles. The resulting PLG-PLE/rGAPDH microparticles were then intraperitoneally administered in grouper (*Epinephelus coioides*). We examined the adjuvant effect of PLE peptide on improving protective immunity induced by rGAPDH protein encapsulated into PLG microparticles. Moreover, protective activities were also evaluated after a lethal peritoneal challenge of *V. harveyi*.

## 2. Experimental

### 2.1. Peptide

PLE peptide (GWGSFFKKAAHVGKHVGKAALTHYL) with amidation at its carboxyl terminus was synthesized using solid-phase peptide synthesis and then purified by reverse-phase high-performance liquid chromatography (RP-HPLC) [27]. The purity (>90%) of the resulting peptide was confirmed by analytical HPLC, and its molecular mass (2.7 kDa) was determined by mass spectrometer (MS) [27]. Synthetic PLE peptide was lyophilized by an FD-5030 freeze dryer (Panchum) for storage at −20 °C and reconstituted in phosphate-buffered saline (PBS, pH 7.4) for use in the present study.

### 2.2. Fish

Orange-spotted groupers (*Epinephelus coioides*), weighing on average 50 g, were purchased from a disease-free farm in southern Taiwan. Fish were cultivated in FRP (fiberglass reinforced plastics) tanks supplied with filtered and aerated regular sea water. Fish were fed with commercial dry pellets twice a day, and their health status was monitored every day. All administrations were reviewed and approved by the Institutional Animal Care and Use Committee, National Pingtung University of Science and Technology.

### 2.3. Bacterial Genomic DNA

The *V. harveyi* BCRC13812 strain was purchased from the Bioresource Collection and Research Center (BCRC), Hsinchu, Taiwan, and was grown in trypticase soy broth (TSB, Difco) with 2% NaCl at 25 °C for 18 h. A total of 1 × 10^8^ bacteria was incubated with lysis buffer (1% sodium dodecyl sulfate, 10 mM Tris-HCl (pH 8.0), 10 mM EDTA, 40 mM NaCl and 100 µg/mL proteinase K) for 1 h at 37 °C, and their DNA was extracted by phenol-chloroform extraction and ethanol precipitation, as described previously [28]. The resulting pellet was incubated with 50 µg/mL RNase A (DNase-free) for 1 h at 37 °C and purified again by phenol-chloroform extraction and ethanol precipitation. After drying out, the DNA pellet was resuspended in distilled water and stored at −20 °C until use.

### 2.4. Expression of Recombinant GAPDH (rGAPDH)

The GAPDH gene (996 bp) of *V. harveyi* (GenBank Accession Number DQ184650.1) was amplified by polymerase chain reaction with a pair of specific primers (forward primer: 5'-CGCGGATCCATGACTATCAAAGTAGGTAT-3' and reverse primer: 5'-CTCCTCGAGCTTAGAGATGTGAGCGATTAG-3'). The forward and reverse primers, respectively, contained *Bam*H I and *Xho* I sequences (underlined). Briefly, 100 ng of *V. harveyi* genomic DNA prepared as mentioned above was incubated with 2 U of Expand high fidelity DNA polymerase (Roche in a 100-µL reaction mixture containing 10 mM Tris-HCl (pH 8.3), 50 mM KCl, 2.5 mM MgCl_2_, 10 µM dNTPs and 1 µM primers. The PCR program was made up of an initial melting at 95 °C for 5 min followed by 30 cycles of amplification, which consisted of incubations at 95 °C for 1 min, 52 °C for 1 min and 72 °C for 1 min. The amplified GAPDH fragment was digested with restriction enzymes *Bam*H I and *Xho* I (Toyobo) and then inserted into the *Bam*H I/*Xho* I sites of plasmid pET24a. The resulting recombinant plasmid was transformed to BL21 (DE3) *E. coli* (Yeastern Biotech). The cloned gene was sequenced by PRISM cycle sequencing systems (ABI) and compared with the previously reported GAPDH gene (GenBank Accession Number DQ184650.1). The histidine-tagged rGAPDH was expressed by induction with 1 mM IPTG (isopropyl-β-d-thiogalactopyranoside) for 4 h at 37 °C. Induced bacteria were obtained by centrifugation at 3,000 ×*g* for 10 min and resuspended in the denaturing lysis buffer (300 mM KCl, 50 mM KH_2_PO_4_, 5 mM imidazole, 6 M urea, pH 8). After incubation on ice for 10 min, bacteria were lysed by repeated freeze/thawing followed by ultrasonication. The supernatant was collected by centrifugation at 13,000 rpm for 20 min at 4 °C and filtered through 0.22-µm disk filters (Millipore). The resulting filtrate was applied into a column with Profinity^TM^ IMAC Ni-charged resin (Bio-Rad) and the rGAPDH was purified by metal chelate affinity according to the manufacturer’s instructions. The resulting purified rGAPDH protein was then dialyzed against PBS to remove urea. The antigenicity of the purified rGAPDH protein was analyzed by western blotting with the use of *V. harveyi*-infected grouper sera.

### 2.5. Infected Grouper Sera

Antisera collected from grouper three weeks after they were peritoneally infected with 1 × 10^4^ CFU of *V. harveyi* (BCRC13812) were used as *V. harveyi*-infected grouper sera in the present study.

### 2.6. Rabbit IgG against Grouper Immunoglobulin

Grouper immunoglobulin (GIg) from the whole serum of grouper was purified using the protein A sepharose column (Bio-Rad) [29]. Anti-GIg sera were collected from rabbits subcutaneously injected twice at a 14-day interval with purified GIg (0.3 mg/rabbit) emulsified with Freund’s adjuvant (Sigma). The IgG fraction in the anti-GIg rabbit sera was purified using the protein A sepharose affinity column (Bio-Rad) and then used as a tool to detect grouper serum Ig in the immunoassays.

### 2.7. Microparticle Preparation

In this study, PLG microparticles containing rGAPDH alone or rGAPDH plus PLE were fabricated by the water/oil/water double emulsion solvent evaporation technique, as described previously [30,31], with minor modifications. Briefly, 20 mL of a 10% solution of 50:50 PLG (Sigma) in dichloromethane (Sigma) was mixed with 2 mL of a rGAPDH solution (5 mg/mL) or a solution containing rGAPDH (5 mg/mL) plus PLE (0.5 mg/mL) by using a PRO200 homogenizer (PRO Scientific) equipped with 10 mm × 150 mm generator at 10,000 rpm for 10 min to produce a water/oil emulsion. The resulting emulsion was further homogenized with 20 mL of a 2% polyvinyl alcohol (Sigma) solution at 10,000 rpm for 10 min to generate a stable water/oil/water emulsion. The water/oil/water emulsion was then stirred for 18 h at room temperature (RT) and pressurized to promote solvent evaporation and the formation of PLG-rGAPDH or PLG-PLE/rGAPDH microparticles in a laboratory fume hood. These microparticles were collected by centrifugation at 4,000 ×*g* for 30 min, washed three times with distilled water to remove non-entrapped protein/peptide and then lyophilized by an FD-5030 freeze dryer (Panchum) for storage at −20 °C. The particle morphology was inspected using a S3000N scanning electron microscope (Hitachi), and the particle size was determined by an N5 submicron particle size analyzer (Beckman Coulter), as before [30,31].

### 2.8. Entrapment Efficiency

The entrapment of protein and/or peptide in PLG microparticles was then measured as follows. A total of 5 mg of PLG-rGAPDH or PLG-PLE/rGAPDH microparticles was first dissolved in 500 μL of 0.1 M NaOH with 2.5% SDS to extract the encapsulated protein/peptide, as described previously [30,31]. After centrifugation at 4,000 ×*g* for 10 min, the content of protein and/or peptide in the supernatant was assessed with the BCA protein assay (Pierce) and compared with BSA standards and adjusted against empty PLG microparticles. Based on this result, the ratio (w/w) of protein and/or peptide entrapped per dry weight of microparticles was determined, and the entrapment efficiency (%) was expressed as the ratio of the actual protein and/or peptide entrapment to the theoretical protein and/or peptide entrapment [30,31]. All measurements were performed in triplicate on samples from different batches.

### 2.9. In Vitro Release Study

A total of 5 mg of PLG-rGAPDH or PLG-PLE/rGAPDH microparticles was suspended in 1 mL of PBS (pH 7.4) with 0.02% sodium azide and shaken at 25 °C in 1.5 mL microfuge tubes. Every six days, 1 mL of supernatant was sampled by centrifugation at 4,000 ×*g* for 30 min and an additional 1 mL of fresh PBS was immediately added to the microfuge tubes in order to incubate as before. The collected samples on Days 6, 12, 18, 24 and 30 were neutralized and analyzed by 18% SDS-PAGE to determine if protein and/or peptide still released from PLG microparticles [31]. 

### 2.10. Immunization

The *in vivo* immunization experiments were carried out in orange-spotted grouper (*Epinephelus coioides*). Four groups of 75 fish each were intraperitoneally injected twice at a 3-week interval with PBS, 10 μg of soluble rGAPDH alone, 10 μg of PLG-rGAPDH microparticles or 10 μg of PLG-PLE/rGAPDH microparticles. During the immunization schedule (12 weeks), the specific anti-*Vibrio* immune responses, including serum responses and lymphocyte proliferation, were analyzed by the following immunoassays.

### 2.11. Antigenic Specificity in Grouper Sera

Three weeks after boosting (the sixth week), the antigenic specificity of the immunized fish sera was analyzed by western blot analysis [30,31]. *V. harveyi* lysate (15 μg/well) was separated by 12% SDS-PAGE and electrophoretically transferred to a polyvinylidene difluoride (PVDF) membrane (Millipore). After blocking, strips of the membrane were cut and probed with sera from fish immunized with PLG-PLE/rGAPDH microparticles, PLG-rGAPDH microparticles, soluble rGAPDH alone or PBS for 1 h at 37 °C. The process with *V. harveyi*-infected fish serum was also conducted. The subsequent color development was processed, as described previously [30,31].

### 2.12. Anti-V. harveyi Serum Titer Assay

Following immunization, fish sera were collected every three weeks, and their serum titers were measured by using ELISA, as described previously [31], with minor modifications. Flat-bottomed 96-well polystyrene microplates (Nunc) were coated with 100 µL/well of *V. harveyi* lysate (10 µg/mL) in 0.1 M carbonate/bicarbonate buffer (pH 9.4) and incubated overnight at 4 °C. Each well was then washed with PBS and blocked with blocking buffer (PBS containing 5% BSA). Samples of 1:50 diluted serum in serial dilution were added to the wells (50 µL/well) and incubated for 1.5 h at 37 °C. After three washes with PBST (PBS with 0.05% Tween 20), the wells were incubated with 1:1000-diluted rabbit anti-grouper immunoglobulin (GIg) IgG for 1 h at 37 °C. PBST washes were carried out again, and in each well was incubated 50 µL of biotinylated goat anti-rabbit IgG antibody (Zymed) diluted in the blocking buffer (1:3000) for 1 h at 37 °C. After washing with PBST, 50 µL/well of streptavidin: peroxidase (1:3000 dilution) were added to incubate for 1 h at room temperature. Color development and serum titer determination were then performed, as described previously [31].

### 2.13. Lymphocyte Proliferation Assay

Like the spleen in mice or humans, the head kidney is an important lymphoid organ in fish. Therefore, in the present study, lymphocyte proliferation in the grouper head kidney in response to GAPDH-containing *V. harveyi* lysate was analyzed to evaluate whether protective cell-mediated immunity was induced. Following immunization, three fish per group were sacrificed every three weeks to obtain lymphocytes of head kidneys via gradient isolation by Ficoll-Paque™ Plus (GE Healthcare) under sterile conditions [31]. The lymphocytes were then cultured in triplicate in 96-well culture plates at a concentration of 1 × 10^6^ cells per well in 200 µL of L-15 culture medium (CM). The cells in each well were stimulated with 20 µg/mL of *V. harveyi* lysate and incubated for 72 h at 25 °C. CM-treated cultures were also conducted to use as controls. The lymphocyte proliferation induced by *V. harveyi* lysate was monitored by using the BrdU (5-bromo-2'-deoxyuridine) Colorimetric Cell Proliferation ELISA Kit (Roche), according to the manufacturer’s instructions [30,31]. Finally, the stimulation index (SI = OD_450_ values from *V. harveyi* lysate-treated cultures/OD_450_ values from CM-treated control cultures) of each group was calculated as described previously [31] and expressed as the mean ± SD.

### 2.14. Bacterial Challenge

Nine weeks after boosting (the 12th week), four groups of 60 fish each were challenged with an intraperitoneal injection of 6 × 10^6^ CFU of *V. harveyi* (BCRC13812 strain) in order to verify whether the induced immune responses could protect fish from *V. harveyi* infection. After the challenge, fish were observed daily for an additional 28 days, and deaths were recorded as they occurred. The survival rate (number of surviving fish after challenge/number of tested fish in each group) in each group was calculated [31].

### 2.15. Statistical Analysis

The data in the present study were statistically analyzed as follows, according to previous studies [30,31]. The particle size and entrapment efficiency of PLG microparticles from different batches were statistically compared using one-way ANOVA. Serum immunoglobulin titers in grouper were transformed logarithmically to attain normality. Log_10_ antibody titers and SI values of different immunization groups were statistically compared using the nested design. The survival rates of different groups were analyzed by the chi-square test. A *p*-value of less than 0.05 was considered a statistically significant difference.

## 3. Results

### 3.1. Antigenicity of E. coli-Based Purified rGAPDH

**Figure 1:**
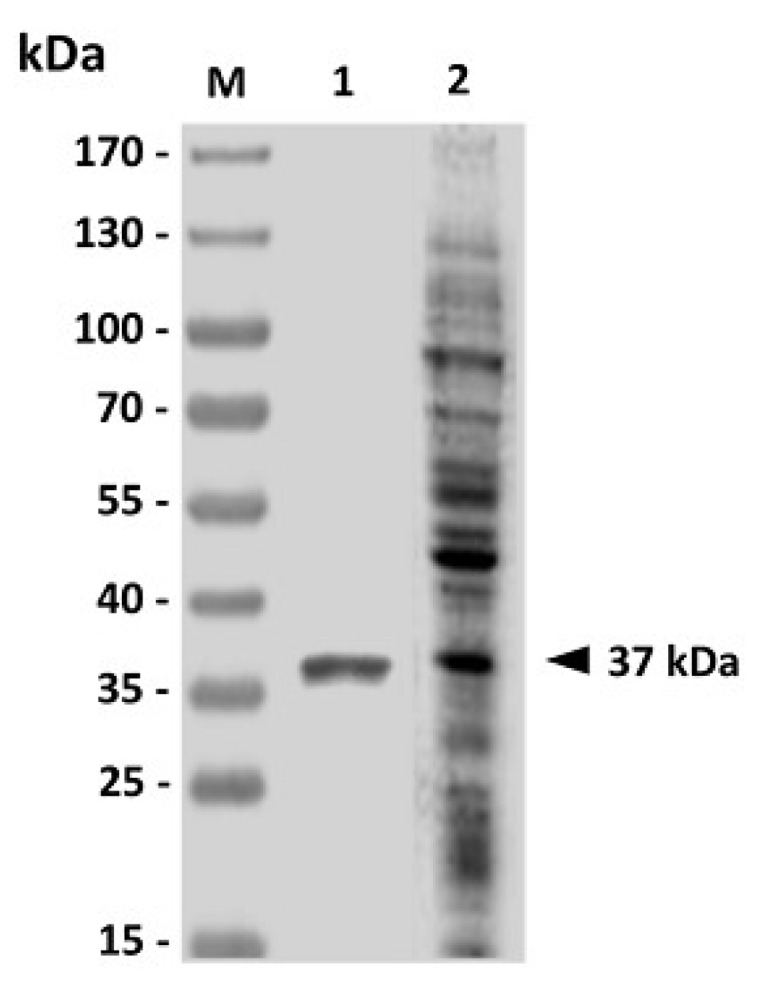
Analysis of purified recombinant GAPDH (rGAPDH) by western blotting. Purified *E. coli*-based rGAPDH protein (Lane 1) was analyzed with *V. harveyi*-infected grouper sera, which could recognize *V. harveyi* lysate (Lane 2). Standard protein markers (Lane M) are shown on the left.

After sequencing, no substitution was found in the cloned GAPDH sequence (data not shown) in comparison with the previously reported GAPDH gene (GenBank Accession Number DQ184650.1). Western blot using *V. harveyi*-infected sera was performed to analyze the purified rGAPDH protein. The *V. harveyi*-infected sera, which recognized *V. harveyi* lysate (Figure 1, lane 2), identified a specific protein band corresponding to the predicted molecular weight (37 kDa) for the rGAPDH protein (Figure 1, Lane 1). 

### 3.2. Characteristics of PLG Microparticles

**Figure 2:**
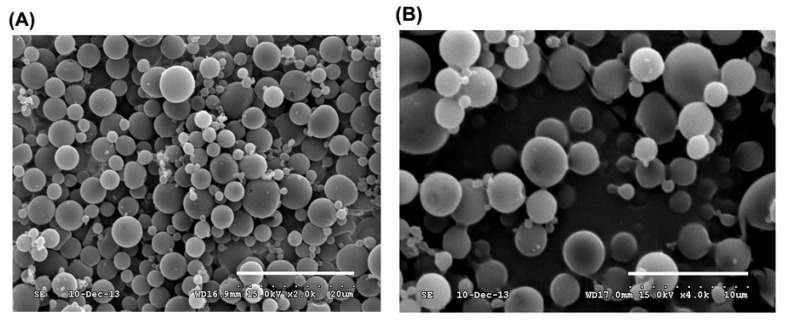
Scanning electron micrographs of PLG-encapsulated microparticles. poly(Lactide-co-glycolide) (PLG)-pleurocidin (PLE)/rGAPDH microparticles (**A**) and PLG-rGAPDH microparticles (**B**) can be seen as spherical particles with a smooth surface (the bar represents 20 μm).

**Figure 3:**
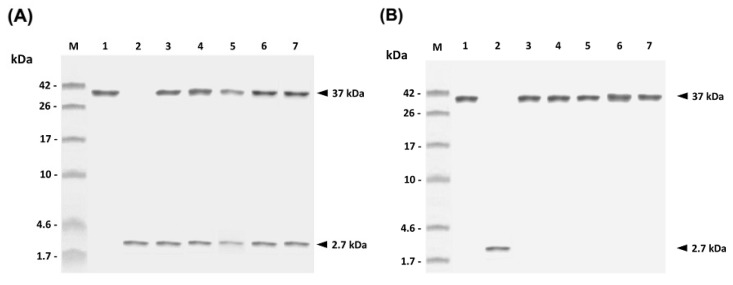
*In vitro* release of rGAPDH or PLE peptide from PLG microparticles. The rGAPDH (Lane 1), PLE peptide (Lane 2), as well as released samples of PLG-PLE/rGAPDH microparticles (**A**) and PLG-rGAPDH microparticles (**B**) on Days 6 (Lane 3), 12 (Lane 4), 18 (Lane 5), 24 (Lane 6) and 30 (Lane 7) were analyzed by 18% SDS-PAGE. Standard protein markers (Lane M) are shown at the left.

rGAPDH alone and rGAPDH mixed with synthetic PLE peptide were respectively encapsulated into PLG microparticles. The resulting PLG-rGAPDH and PLG-PLE/rGAPDH microparticles were then analyzed. The morphology of microparticles was first inspected by scanning electron microscopy. Both PLG-PLE/rGAPDH and PLG-rGAPDH microparticles showed a uniform population of spherical particles with a smooth surface (Figure 2). A particle size analyzer was further used to determine the particle size. Different PLG-PLE/rGAPDH preparations showed a mean diameter ranging from 3.21 to 6.27 µm, and their entrapment efficiency for rGAPDH plus PLE ranged from 72% to 83%, with a mean encapsulation efficiency of 77% (Table 1). In each preparation of PLG-PLE/rGAPDH microparticles (10 mg of rGAPDH and 1 mg of PLE were used), therefore, the total amount of 7.7 mg rGAPDH and 0.77 mg PLE was actually encapsulated into PLG microparticles. The cumulative release of rGAPDH and PLE during the course of a 30-day period was 82% of the total protein/peptide load (data not shown). Therefore, 6.31 mg of rGAPDH and 0.63 mg of PLE were released from the original microparticle. In addition, another PLG microparticle vaccine, PLG-rGAPDH microparticles, was also prepared in the present study. As shown in Table 1, the mean diameter of different PLG-rGAPDH batches ranged from 2.84 to 6.02 µm, and the entrapment efficiency for rGAPDH was from 69% to 78%, with a mean encapsulation efficiency of 74%. In each preparation of PLG-rGAPDH microparticles (10 mg of rGAPDH were used), the total amount of 7.4 mg rGAPDH was actually encapsulated into PLG microparticles. The cumulative release of rGAPDH during the course of a 30-day period was 80% of the total protein load (data not shown). Therefore, 5.92 mg of rGAPDH was released from the original microparticle. In order to determine if the prepared microparticles released protein and/or peptide, 18% SDS-PAGE was undertaken to examine released samples collected on different days (Figure 3). Results showed that the *in vitro* release of rGAPDH plus PLE peptide (Figure 3A) or rGAPDH alone (Figure 3B) from PLG microparticles could be maintained during the course of a long 30-day period. 

**Table 1:** Particle size and entrapment efficiency of PLG-encapsulated microparticles.

PLG-PLE/rGAPDH microparticles		
Batch	Particle size (μm) ^a^	Entrapment efficiency (%) ^b^
1	4.62 ± 1.38 ^c^	75 ± 19 ^d^
2	6.27 ± 2.02 ^c^	83 ± 20 ^d^
3	3.21 ± 1.65 ^c^	72 ± 16 ^d^
**PLG-rGAPDH microparticles**		
**Batch**	**Particle size (μm)**	**Entrapment efficiency (%)**
1	6.02 ± 2.14 ^e^	78 ± 28 ^f^
2	3.63 ± 1.84 ^e^	74 ± 21 ^f^
3	2.84 ± 1.27 ^e^	69 ± 18 ^f^

^a^ The particle size in diameter was measured and expressed as the mean ± SD; ^b^ entrapment efficiency was expressed as the ratio of the actual protein entrapment to the theoretical protein entrapment, as described in the Experimental Section; ^c,d,e,f^ a significant difference (*p* < 0.05) exists between different batches with different superscript letters.

### 3.3. GAPDH-Specific Serum Response in Immunized Grouper

**Figure 4:**
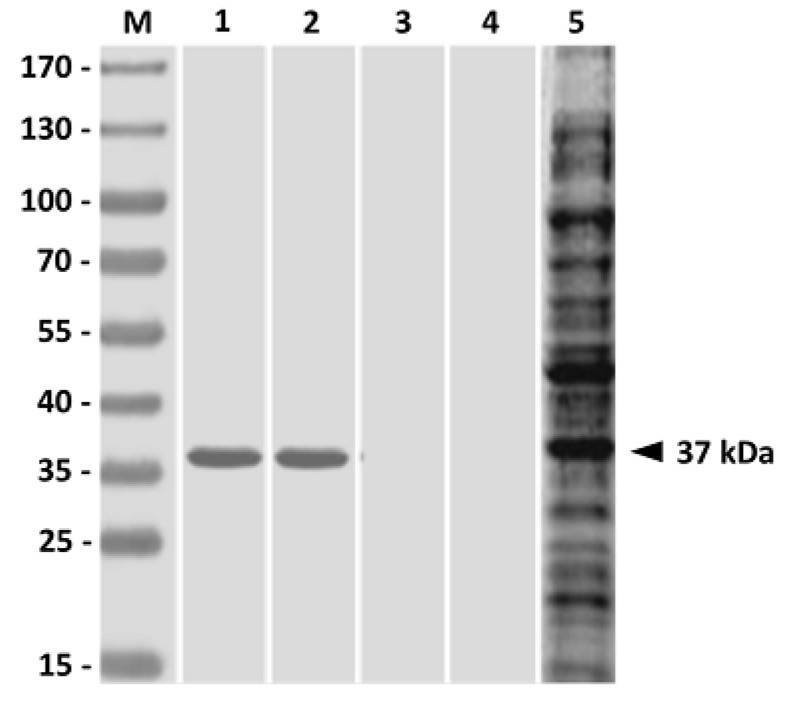
Antigenic specificity in immunized grouper sera. Three weeks after boosting, grouper sera were collected to analyze their antigenic specificity. *V. harveyi* lysate was probed with sera from grouper peritoneally immunized with PLG-PLE/rGAPDH microparticles (Lane 1), PLG-rGAPDH microparticles (Lane 2), rGAPDH alone (Lane 3) or PBS (Lane 4). The *V. harveyi*-infected grouper serum was also conducted (Lane 5). Standard protein markers (Lane M) are shown on the left.

**Figure 5:**
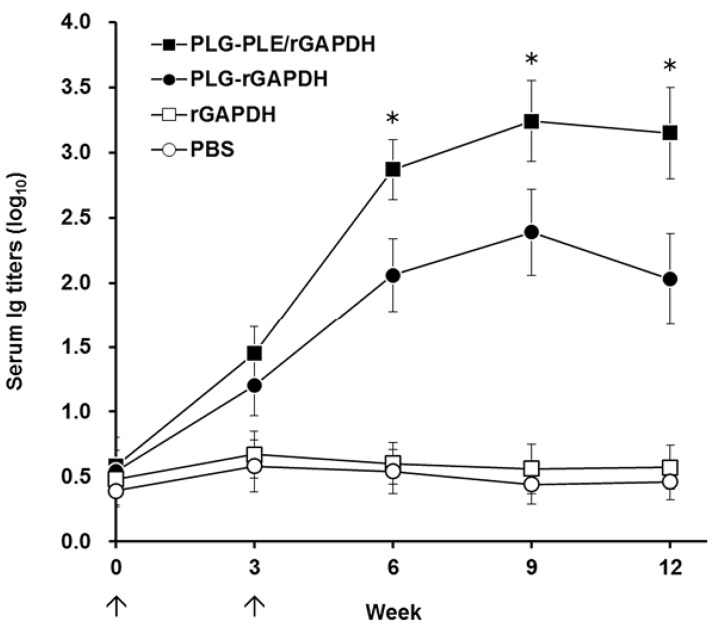
Serum immunoglobulin (Ig) titers against *V. harveyi* in immunized grouper. Four groups of grouper were intraperitoneally immunized twice (↑) with PLG-PLE/rGAPDH microparticles (■), PLG-rGAPDH microparticles (●), rGAPDH alone (□) or PBS (○). Sera were collected from three fish per group every three weeks, and their anti-*V. harveyi* serum Ig titers were determined by ELISA. Results were presented as the mean of log_10_ titers ± SD. * *p* < 0.05 when comparing the PLG-PLE/rGAPDH group to the PLG-rGAPDH group.

The ability of PLG microparticles to trigger humoral immunity against *V. harveyi* in grouper was subsequently evaluated. Western blot studies of fish sera obtained three weeks after boosting showed that both PLG-rGAPDH and PLG-PLE/rGAPDH microparticles resulted in the production of grouper serum antibodies against the native GAPDH protein in *V. harveyi* lysate (Figure 4, Lanes 1 and 2). However, sera from grouper immunized with rGAPDH alone or PBS did not recognize any proteins in *V. harveyi* lysate (Figure 4, Lanes 3 and 4). Therefore, intraperitoneal immunization with rGAPDH in grouper could elicit a specific serum response to the native GAPDH protein in *V. harveyi* lysate only when rGAPDH protein was encapsulated with the PLG polymer, but not in its soluble form. In addition, every three weeks, the specific anti-*V. harveyi* serum titers in grouper were determined by ELISA (Figure 5). Intraperitoneal immunization with PLG-PLE/rGAPDH or PLG-rGAPDH microparticles was able to durably keep high serum titers, up till the 12th week. More importantly, three weeks after boosting (the sixth week), grouper serum titers induced by PLG-PLE/rGAPDH microparticles were significantly higher (*p* < 0.05, nested design) than those of PLG-rGAPDH microparticles (Figure 5). However, grouper immunized with soluble rGAPDH alone or PBS displayed little, if any, anti-*V. harveyi* serum titers (Figure 5). Therefore, the presence of PLE peptide improved the grouper anti-*V. harveyi* serum response induced by rGAPDH encapsulated in PLG microparticles. 

### 3.4. GAPDH-Specific Lymphocyte Proliferation in Immunized Grouper

**Figure 6:**
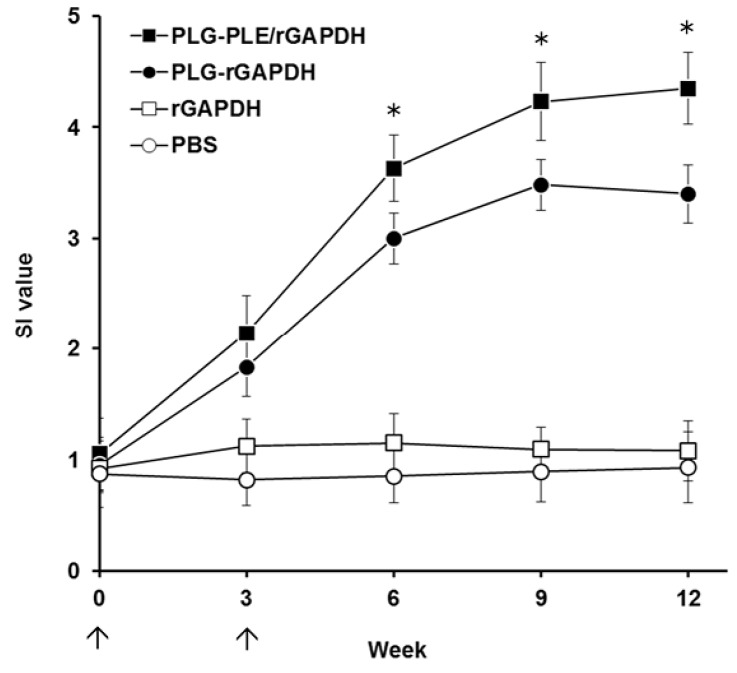
Proliferation responses against *V. harveyi* in immunized grouper. Four groups of grouper were intraperitoneally immunized twice (↑) with PLG-PLE/rGAPDH microparticles (■), PLG-rGAPDH microparticles (●), rGAPDH alone (□) or PBS (○). After immunization, *V. harveyi* lysate-stimulated head kidney lymphocytes were prepared from three fish per group every three weeks, and their subsequent proliferation responses were analyzed and expressed as stimulation index (SI) values. Results were presented as the mean of SI values ± SD. * *p* < 0.05 when comparing the PLG-PLE/rGAPDH group to the PLG-rGAPDH group.

Every three weeks, head kidney lymphocytes stimulated with *V. harveyi* lysate were prepared from different groups of fish, and their subsequent proliferation responses were analyzed and expressed as SI values (Figure 6). Both PLG-PLE/rGAPDH and PLG-rGAPDH microparticles, respectively, gave rise to sustained lymphocyte proliferation for twelve weeks. Three weeks after boosting (the sixth week), PLG-PLE/rGAPDH microparticles elicited significantly higher SI values (*p* < 0.05, nested design) than PLG-rGAPDH microparticles did (Figure 6). However, administration with soluble rGAPDH alone or PBS induced little lymphocyte proliferation in grouper (Figure 6). The synthetic PLE peptide, therefore, enhanced grouper anti-*V. harveyi* lymphocyte proliferation elicited by immunization with PLG-encapsulated rGAPDH protein.

### 3.5. Protection against V. harveyi Challenge

**Figure 7:**
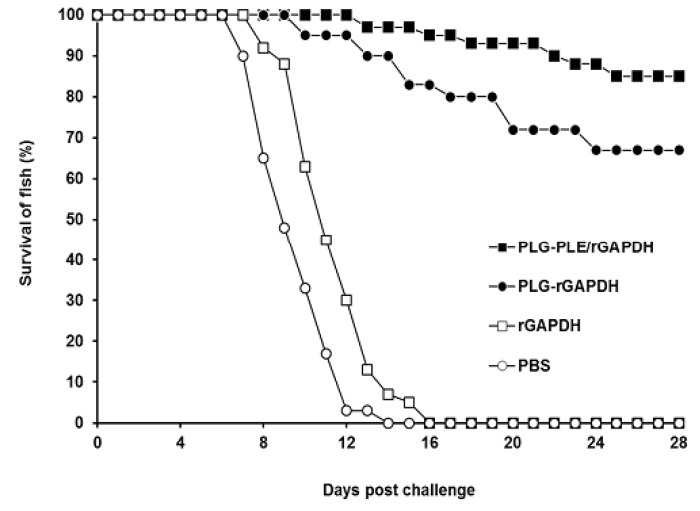
Survival of immunized grouper after a lethal *V. harveyi* challenge. Grouper were intraperitoneally immunized twice with PLG-PLE/rGAPDH microparticles (■), PLG-rGAPDH microparticles (●), rGAPDH alone (□) or PBS (○). Nine weeks after boosting, four groups of 60 groupers each were intraperitoneally infected with 6 × 10^6^ CFU of *V. harveyi* (BCRC13812 strain). Fish were observed daily for an additional month (28 days), and the final survival rates were calculated. * *p* < 0.05 when comparing the PLG-PLE/rGAPDH group to the PLG-rGAPDH group.

We then determined whether PLG microparticle vaccines could confer effective protection in grouper. Nine weeks after boosting (the 12th week), all groups of 60 groupers each were intraperitoneally challenged with 6 × 10^6^ CFU of *V. harveyi* (BCRC13812 strain). Fish were observed daily for an additional month (28 days) and the survival rates were recorded (Figure 7). All fish administrated with soluble rGAPDH alone or PBS died within 16 days after challenge and displayed no protection against the experimental challenge. Forty fish immunized with PLG-rGAPDH microparticles survived during the challenge study and showed a protection rate of 67%. However, in the group of fish immunized with PLG-PLE/rGAPDH microparticles, only nine fish died during the challenge study. Thus, intraperitoneal immunization with PLG-PLE/rGAPDH microparticles in grouper resulted in the highest survival rate (85%), which was significantly higher (*p* < 0.05, chi-square test) than that of the PLG-rGAPDH group (67%) (Figure 7). Therefore, the addition of the peptide adjuvant, PLE, into the vaccine formulation provided a substantial advance in protection against *V. harveyi*.

## 4. Discussion

Most modern vaccines based on purified proteins are poorly immunogenic and, therefore, require effective adjuvants to aid them in eliciting strong immune responses [9,10,11]. Significant information obtained recently indicates that future investigations on the development of fish vaccines will have to include efficacious adjuvants capable of enhancing immunity and protection against aquacultural infectious diseases [9]. As novel peptides, especially AMPs, have been demonstrated to activate both innate and adaptive immune responses, there has been an intense interest in the development of these peptides as powerful adjuvants in numerous studies [10]. In the present study, we have demonstrated that the addition of PLE peptide can improve the immunogenicity of *E. coli*-based rGAPDH protein encapsulated into PLG microparticles to advance long-lasting protective immunity against *V. harveyi* in grouper, indicating the importance of the use of the peptide adjuvant, PLE. 

PLE peptide can have an adjuvant effect in activating and recruiting immune cells to the site for the initiation of innate and adaptive immune responses [18,19]. Thus, improvement in the stability of PLE peptide in animals would be a critical step forward in maintaining the PLE-induced adjuvant effect. The modification by amidation at the carboxyl terminus of PLE in the present study has been demonstrated to exhibit improved proteolytic stability, thereby prolonging its longevity in target animals [22,23]. In addition, the ability of PLG-PLE/rGAPDH microparticles to control the sustained release of PLE peptide (Figure 3A) is an attractive characteristic to substantially prolong the recruiting effect following vaccination in grouper [24,25]. In other words, both carboxyl amidation and PLG encapsulation mutually cooperated to prolong the adjuvant effect of PLE peptide to improve immune responses in grouper (Figure 5 and Figure 6).

In the present study, the peptide adjuvant, PLE, and the rGAPDH protein were encapsulated into PLG microparticles by the double emulsion method. After PLG encapsulation, SDS-PAGE analysis of the released samples collected on different days demonstrated that both PLE and rGAPDH were able to be released from PLG microparticles for at least 30 days (Figure 3A). The lack of major smaller or larger fragments on the SDS-PAGE further indicated the integrity of PLE and rGAPDH during the 30-day release period (Figure 3A). Furthermore, the enhanced long-lasting GAPDH-specific immunity in grouper (Figure 5 and Figure 6) provided notable evidence that the antigenicity of rGAPDH and the adjuvant effect of PLE could be preserved by the preparation procedure of PLG-PLE/rGAPDH microparticles. Therefore, both the encapsulation procedure and the sustained release from microparticles in the present study were not harmful to the antigenicity of rGAPDH and the adjuvant effect of PLE.

An indicative hallmark of an efficacious vaccine used in fish is the ability to induce strong cell-mediated immunity [9]. In the present study, after peritoneal immunization in grouper, we focused much attention on the lymphocyte proliferation response, an activity that has been demonstrated to positively correlate with cell-mediated immunity in our previous studies [28,30,31]. We found that both PLG-PLE/rGAPDH and PLG-rGAPDH microparticles resulted in long-lasting (12 weeks) lymphocyte proliferation in the grouper head kidney (Figure 6). Furthermore, in comparison, PLG-PLE/rGAPDH microparticles produced a significantly higher proliferation response than PLG-rGAPDH microparticles did. These data indicate that the PLE peptide adjuvant truly enhances the long-lasting GAPDH-specific cell-mediated immunity in grouper.

In addition to cell-mediated immunity, enhanced anti-*V. harveyi* titers detected in the grouper serum (Figure 5) following peritoneal immunization with PLG-PLE/rGAPDH microparticles also pointed out that the B cell-mediated humoral response should contribute to the resistance against *V. harveyi*. In other words, PLG-PLE/rGAPDH microparticles elicited mixed Th1/Th2 immune responses against *V. harveyi* in grouper. In the present study, the long-lasting cell-mediated and humoral immune responses induced by PLG-PLE/rGAPDH microparticles protected 85% of grouper against a lethal peritoneal *V. harveyi* challenge and allowed fish to survive for a long period of 28 days after the experimental challenge (Figure 7). In further comparison, PLG-PLE/rGAPDH microparticles elicited a significantly higher protective rate (85%) in grouper than PLG-rGAPDH microparticles (67%). Due to the presence of the PLE peptide adjuvant, the survival rate was therefore increased by 18% in the group of fish immunized with PLG-PLE/rGAPDH microparticles. Immunoprotection against *V. harveyi* induced by GAPDH has been previously described in another fish species, large yellow croakers (*Pseudosciaena crocea*) [6,7]. However, the induced immune responses have produced only partial protective efficacy (40%) against a lethal challenge dose of the virulent strain of *V. harveyi*. Despite differences in fish species and bacterial strains used for *in vivo* studies, the notable vaccine potency observed in the present study indicated that the administration of PLE peptide conferred a substantial adjuvant effect in improving anti-*V. harveyi* protection in grouper induced by rGAPDH encapsulated in PLG microparticles.

## 5. Conclusions

In the present study, we have demonstrated the adjuvant effect of PLE peptide. Furthermore, the sustained release of PLE peptide cannot only improve GAPDH-specific humoral and cell-mediated immune responses, but also induce high protection against *V. harveyi* following peritoneal immunization with PLG-PLE/rGAPDH microparticle in grouper. Therefore, the use of PLE peptide adjuvant in the vaccine formulation provides a valuable approach for the development of efficacious animal vaccines in the future.
